# Detection of Abnormal Respiration from Multiple-Input Respiratory Signals

**DOI:** 10.3390/s20102977

**Published:** 2020-05-24

**Authors:** Ju O Kim, Deokwoo Lee

**Affiliations:** Department of Computer Engineering, Keimyung University, Daegu 42601, Korea; monkey4650@gmail.com

**Keywords:** respiratory status, respiration signal, radar, filter

## Abstract

In this paper, we propose a novel approach for the detection of abnormal signals from multiple respiration signals. An ultrawide-band (UWB) radar was used to acquire respiration signals that represent a distance from the chest to the radar sensor, i.e., shape variation of the chest due to breathing (inhaling or exhaling) activity provides quantitative information (distance values) about respiratory status. Distribution, shape, and variation of values across time provide information to determine respiratory status, one of the most important indicators of human health. In this paper, respiratory status was categorized into two classes, normal and abnormal. Abnormal respiration (apnea in this paper) was emulated by interrupting breathing activity because it is difficult to acquire real apnea from patients in hospital wards. This paper considered two cases, single and multiple respiration. In the first case, a single normal- or abnormal-respiration signal was used as input, and output was the classified status of respiration. In the second case, multiple respiration signals were simultaneously used as inputs, and we focused on determining the existence of abnormal signals in multiple respiration signals. In the case of multiple inputs, filters with varying cut-off frequency were applied to input signals followed by the analysis of output signals in response to the filters. To substantiate the proposed method, experiment results are provided. In this paper, classification results showed 93% of the successful rate in the case of multiple inputs, and results are promising for applications to monitoring systems of human respiration.

## 1. Introduction

Rates or states of respiration are considered one of the most important indicators to evaluate the physical, physiological, and psychological state of human health because breathing activity is the most important and crucial task for humans. According to a significant growth of demand for monitoring human vital signs (or other appropriate indicators that provide information about human health), numerous research activities have been performed in practical areas to yield the comfortable, simple, and accurate measurement of vital signs. Failure of adequate respiration can lead to critical and risky health status in a few minutes, so continuously monitoring respiratory status is crucial and mandatory in clinical fields. To acquire vital signals from a person, there are various types of instruments, but in general, the measuring method is categorized into two classes, contact and noncontact. As both methods utilize data acquired from organs related to breathing activity, instrument sensitivity affects the quality of the acquired respiration data. In contact-based methods, sensing devices (e.g., sensors) are generally attached to the human body to acquire vital signals. Noncontact based methods also employ sensing devices, but those are not attached to the human body. Contact-based methods are usually considered more reliable and accurate than noncontact-based ones. In hospital wards, by using contact-based methods, vital signals are measured on a daily basis, but patients suffer from discomfort or difficulties in the use of the instruments or devices while being measured, especially child patients or burn victims. Thus, contact-based methods have limitations in the long-term use for continuous monitoring, and once measurement starts, movement or other activities except for breathing are not accepted, and this restriction can distress patients or other tested persons. Despite the limitations, contact-based methods are widely employed for clinical evaluation because hospital environments are almost perfectly settled up so that respiration data can be acquired and stored with reliable and stable accuracy. Contact-based methods usually evaluate respiratory status on the basis of the sounds, movements, or temperature of the human body (positions of the parts are usually close to respiratory organs). As breathing activity (inhalation and exhalation) induces variation or movement of respiration-related organs, the technology of respiratory measurement focuses on the movement or variation of those parts where the devices are attached. Airflow-based methods detect respiration status on the basis of the characteristics of inhalation and exhalation. Widely used flow-sensing techniques are differential flowmeters (DF), turbine flowmeters (TF), hot-wire anemometers (HWA), and fiberoptic sensors (FOS). Variations of humidity, velocity, or temperature appear differently in phases of inhalation and exhalation, and the distribution of acquired respiratory data enables us to estimate respiratory rate. Air temperature or volume (or their variations) during exhalation and inhalation or nasal pressure also provides data to be analyzed to monitor respiratory status. To achieve accurate results in airflow-based methods, measurements need to be performed in a controlled environment [1]. In addition to using temperature or volume, movements of the chest or the abdominal wall can also be used to monitor respiratory status, and the device is attached to a position near the chest. Fiberoptic-based measurement employs a photodetector to measure light power that estimates the variation of intensity caused by inhalation and exhalation [2]. To utilize airflow data to predict or indicate respiratory status, high sensitivity of equipment, accurate postprocessing, and perfect experiment setup are required, and to be monitored in a real-time manner, the sensor must always be exposed to inhaling and exhaling activities. Acoustics-based methods utilize respiratory patterns such as variation, loudness, or the frequency spectrum of a measured signal to assess respiratory status with sensors or microphones. Microphones, considered a simple method to implement, are usually located in the front of the neck or nose to acquire breathing sounds during sleep or regular activities. Analysis of the measured respiration signals is carried out in various domains such as frequency, time, *Z*, and wavelet [3]. Acoustics-based methods (usually focusing on the detection and classification of respiration phases) have gained attention as an alternative to airflow-based methods due to their simplicity and convenience [4,5,6]. However, as devices need to be placed close to or attached to the throat, respiration airways, or in front of the nose, or be attached to the neck, the measurement methods are prone to discomfort. In addition to discomfort, if external noise signals are added to the respiration signals, measurement accuracy is degraded, leading to a demand for the preprocessing of acquired signals [7]. Electrocardiogram (ECG) signals have been widely used to measure the electrical activity of the heart from where respiration signals can be separated, and the separated signal is called ECG-derived respiration (EDR) [8]. Once anECG signal is acquired, it consists of the heart rate, respiratory rate, and an additive noise signal, and the mixture of all signals leads to needing to extract respiration data with techniques of signal processing. In contact- or noncontact-based methods, numerous algorithms have been proposed to accurately estimate the respiratory rate from an electrocardiogram (ECG) on the basis of information about chest movements (this is related to heart rate and lung volume) indicating human respiratory status. In ECG-derived methods, approaches to the decomposition of target data were proposed such as component analysis (principal-component analysis (PCA), independent component analysis, kernel PCA, adapted PCA, and canonical CA), decomposition (empirical-mode decomposition, wavelet decomposition), and correlation analysis or learning-based classification [9,10,11,12]. ECG signals are acquired and recorded by the movement of electrodes attached to the chest surface, having relative positions to the heart during respiratory activity. Once the acquisition process starts, P, QRS, and T waves are carefully observed and analyzed. The P wave is associated with right and left atrial depolarization, QRS (called the QRS complex) is associated with right and left ventricular depolarization, and the T wave represents ventricular repolarization. ECG-derived respiration analyzes these waves, and this is a very well known simple method to monitor respiratory status unless motion artefacts are eliminated [13,14]. In optical-fiber-based measurements, the polymeric optical fiber sensitively responds to the applied pressure, enabling to monitor respiratory status and manufacture wearable devices for measurements [15]. If a statistical assumption about respiration signals does not hold in realistic environment (for example, an ICA (independent component analysis)-based method tries to decompose ECG signals into independent components, so statistical independence is assumed between the subcomponents of the ECG signal), it does not guarantee the accurate measurement of respiratory status.

Recently, noncontact (noninvasive)-based respiration measurement has been of interest because of the development of wearable devices and the increasing demand for the convenient and long-term continuous monitoring of the physiological status of patients [16]. Thus, noncontact-based methods are considered an alternative to contact-based ones, practically providing convenience and simplicity to monitor respiratory status because sensing devices do not have to be attached to the human body. Sensing devices are usually at a distant position so that a person can perform their usual activities while real-time and long-term measurement is in process. The range of working distance between device and human body depends on the hardware performance of the device, which is not discussed in this paper. The methods monitor respiratory states on the basis of variations of the morphology, geometry, intensity, or temperature of target areas such as the chest or throat. Although noncontact-based methods provide convenience, a controlled experiment setup is still required, similarly to contact-based ones, because the existence of external noise can degrade measurement performance. Among many noncontact-based methods, radar-based ones are of the most widely used, minimizing discomfort with low cost; their basic principle is to measure the distance between sensor and human body (especially part of the chest) using transmitted and received radar signals. Inhalation and exhalation generate chest-shape variations, leading to a change of distance from the radar to the chest surface and respiratory status can be indirectly estimated. A Doppler radar detects respiratory patterns in addition to measuring respiratory rate, facilitating the real-time monitoring of respiratory status [17]. A combination of vibration and pyroelectric infrared (PIR) sensors is a noncontact-based method; the former detects and measures vibrations and the latter detects thoracic movements resulted from breathing activity. Vibration sensors detect the motion of a target and convert mechanic motions to electrical signals. Both sensors are located at distant positions from the human body, and multimodal signal processing is conducted (wavelet and empirical-mode decomposition) using the periodicity of breathing activity [18]. Doppler radars also measure respiration signals composed of harmonic interference, the movements of any part of human body, clutter noise, etc. Respiration signals are measured and analyzed on the basis of the difference of phases between transmitted and received signals that are represented in harmonic basis. The movement of the nasal part of the human body affects phase changes [19]. A received respiration signal is analyzed in the frequency domain, and respiratory status, such as normal breathing, inhalation, exhalation, or sleep apnea, is estimated for classification. Estimation of respiration in the time domain employs a correlation model to measure respiratory rate [20]. As the Doppler radar measures phase changes to measure the displacement of a chest surface, measurement setup should be carefully carried out so that other activities (called motion artefacts) except for respiration are not measured by the radar. In addition to the noise, the bandwidth of the heartbeat signal overlaps with that of the respiration signal, so concerning the above problems, the classification or separation of respiration signals from motion artefacts is desired. To remove or minimize artefacts, algorithms were proposed to process input signals, such as denoising, signal separation (e.g., blind-source separation), and the decomposition of signals [21]. Optical-imaging systems are another way of noncontact-based monitoring by detecting or observing respiratory patterns such as movements of the chest or thorax during breathing [22]. This method chiefly deals with static or dynamic images so it can track motions induced by breathing activities or track changes of the chest or abdominal surface. Accurate motion tracking (related to breathing activity) is required in optical-imaging-based methods so that the system can register the deformed shape of a body part while removing motion artefacts and determine respiratory status [23]. According to the development of imaging technology, the image-based estimation of respiratory status is considered another popular method. Thermal sensors or images can provide information to monitor respiratory status, and a commercial digital camera with low cost can be used. Detection of the warmest and coolest area in the human face is carried out with high accuracy, and measurement of thermal changes is performed with high accuracy. Thermal image (or infrared thermography)-based methods monitor respiration profiles by measuring thermal variations and hyperventilation, detecting or tracking target areas related to breathing activity (e.g., nose, throat, or chest) to detect unusual respiratory patterns [24]. There is no absolute solution to achieve to most accurately register respiratory status. However, with significant progress in measuring technology, respiration signals play a key role in the prediction and estimation of human health. However, as measurement convenience and simplicity, respiration-based estimation of health status is a crucial part in healthcare fields. Abnormal respiration can be an indicator of a chronic condition such as physiological instability, pneumonia, chronic obstructive pulmonary disease (COPD), or chronic obstructive lung disease, so real-time and continuous long-term monitoring with a relatively low cost of respiration measurement is very important in practice. In the course of the respiration-based estimation of health status, there are two main approaches. One is to measure the respiratory rate, and the other is to detect and classify respiratory patterns. Conventional works focus more on the former, and recent work has begun to focus on the latter.

In this paper, we chiefly focus on the detection and classification of respiratory status that is categorized into two classes, normal and abnormal respiration. Previous work on the processing and analysis of respiration signals has chiefly dealt with measuring the respiratory rate rather than providing information about respiratory status. Using an ultrawide-band (UWB) radar, exhalation and inhalation generate a waveform that represents the distance between radar and chest surface. Distance varies with exhalation and inhalation, and different respiration statuses generate different waveforms. This paper chiefly deals with two cases: single- and multiple-respiration signals. The former used one respiration signal and the latter multiple (two or three) signals as input. In the case of the single input, the received signal was analyzed in the *Z*-domain given one known respiration datum, particularly using pole and zero positions. In the cases of multiple inputs, under the assumption that inputs are simultaneously received, the proposed approach detected the existence of abnormal respiration. The proposed approach, to summarize, aimed at detecting and classifying respiratory status. The proposed approach classifies respiration status considering two main environments. The first is classifying respiratory status using a single-input signal. The second uses multiple (more than two)-input respiration signals as inputs; then, inputs containing at least one abnormal respiration signal are distinguished from those that do not contain abnormal signals. In a real environment, such as vehicles (driver and passengers in a car), this leads to increased necessity to propose an approach for the detection of abnormal respiration from multiple inputs. To substantiate the proposed approach, real respiration signals were acquired using a radar sensor, and respiratory status was classified. The rest of the paper is organized as follows. Section 2 describes the respiration acquisition using a radar sensor. Section 3 presents the proposed approach for the detection of abnormal respiration in the case of single and multiple inputs. Section 4 shows the experiment results of respiratory-status classification, and concludes this paper.

## 2. Acquisition of Respiration Signals

**Figure 1:**
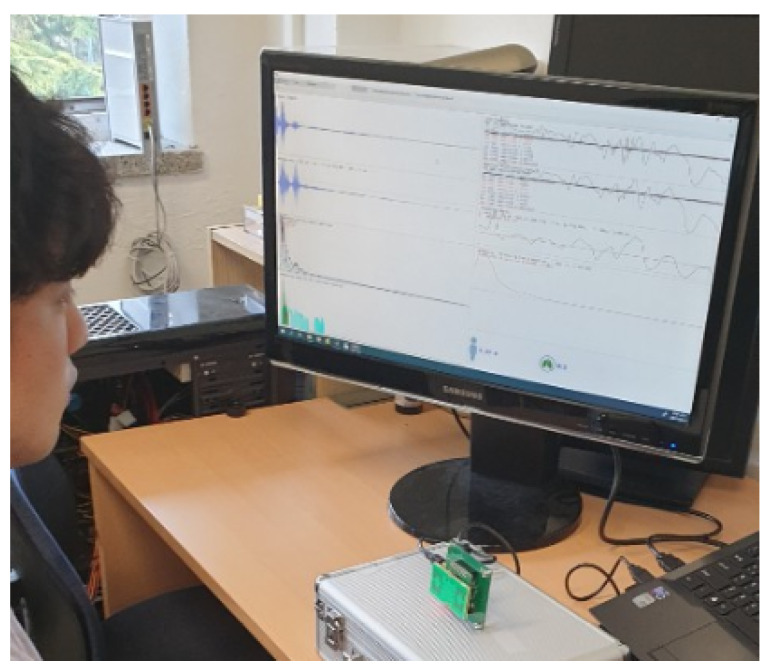
Experiment setup for signal acquisition.

This work is on the detection of abnormal respiration signals in more practical environments rather than environments in hospital wards. In other words, the acquisition of respiration signals is carried out on the basis of usual conditions in daily-life spaces (house or study rooms). In previous or currently existing works on respiration signal, it chiefly deals with the accurate measurement of the respiratory rate because it (or the number of inhalations and exhalations) is a very important indicator to evaluate human health. However, accuracy highly depends on hardware performance. However, we focused on more practical cases, such as outdoor and indoor environments (e.g., inside a car) rather than the environment in hospital wards. We chiefly focused on monitoring the state of human respiration, and investigated whether abnormal respiration existed so that pre-emptive actions could be taken in practical conditions. We dealt with two cases. First, we used two respiration signals to determine the abnormal status of a single respiration (Section 3.1 details this case). In particular, given one respiration signal whose status is already known, it is investigated whether the next input respiration is normal or abnormal. In this case, we investigated the pole-zero plot of the data transformed to the *Z* domain, and the status of the next respiration affected the distribution of the locations of poles and zeros. Second, we used multiple respiration signals simultaneously; then, the existence of abnormal respiration was detected using a high pass filter by varying cut-off frequency (Section 3.2 details this case). This paper utilized an ultrawide-band (UWB) radar sensor to acquire the respiration signal, and the received waveform reflected the shape variation of the chest surface. The UWB radar has also been used for gesture or motion recognition or detection because it transmits and receives high-frequency signals with high speed and resolution, and low-power consumption [25]. Novelda X4 (Novelda, Oslo, Norway) shows better performance than a traditional radar sensor due to its compact size, efficient power consumption, accuracy, and speed. It has better performance than that of a microwave radar (e.g., Doppler) because it is robust to interference and has high resolving power. In general, if a UWB radar is used, the frequency band is in the range of 3.1–10.6 GHz. This frequency band is robust to interference, has high-speed communication, and coverage is from 2 to 10 m. According to the policy of distribution of the frequency band, 3.1–4.8 GHz (low band) and 7.2–10.2 GHz (high band) are assigned; this work used 8.2 GHz for the experiments. This was a single small chip that detected small changes or movements of a chest surface during breathing. Novellda X4 is also considered suitable for monitoring heart rate in hospitals. In areas of motion or gesture recognition, image-based algorithms have gained more attention than other ones. However, image-based algorithms that usually acquire data using a photo or video camera have drawbacks in environments with ambient light, low light, fog, smoke, etc. UWB radars are robust to noise factors as those mentioned above, particularly having accurate measurements in low-light conditions (this is important because respiration is monitored during sleep in medical fields), making it suitable for measuring human respiration [26]. The sensor was located about 30–50 cm from the chest surface. Shape variations come from the movement of the chest surface with breathing. Exhalation and inhalation generate distance variation between radar sensor and chest surface. The present work with the noncontact-based measurement of respiration employed UWB EVM (BR-EVM-2000) Novellda X4, and signal acquisition was done by adults in a real environment (Figure 1).

Once the radar detects a movement of the chest surface from inhalation and exhalation, the distance between radar and chest surface is generated as a waveform that can provide crucial information about respiratory status. In this work, we aimed at classifying respiratory status into two categories, normal and abnormal respiration. Normal respiration generates a waveform that is similar to sinusoidal; in abnormal respiration, the waveform shape is different than that of the normal. In this paper, apnea is considered abnormal respiration, and other kinds of abnormal-respiration signals are not discussed in the paper because it is difficult to acquire diverse abnormal-respiration signals unless they are collected in hospital wards. Normal-respiration signals are acquired in the following conditions:

- stable pose without any other activities,
- breathing with speaking activities, and
- breathing with small movements (walking or after exercise).

**Figure 2:**
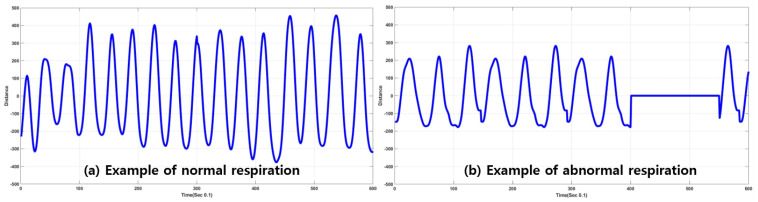
Ultrawide-band (UWB) radar detect motion of chest surface and generates quantitative data that describe distance variation during exhalation and inhalation.

Abnormal respiration in this paper is generated by pausing breathing for a few seconds to emulate apnea because real apnea/abnormal respiration is difficult to acquire. Respiration data are represented as a waveform for one minute. Sampling rate was ten per second, so 600 points were sampled for one minute. Once respiration signals were received, and waveforms were generated, analysis and extraction of respiratory status were conducted for detection and classification. Figure 2 shows examples of plots depicting distances varying by inhalation and exhalation. In the case of normal respiration, the plot is very similar to the sinusoidal form, and it also seemed that the waveform showed gradual (or smooth) variation of distance values between radar and chest. However, in the case of abnormal respiration, there existed sharp variation (or high-frequency components) in the acquired data. The frequency components of the acquired signal provided information to classify respiration status. Thus, Fourier transform or short-term Fourier transform (STFT) can be employed to analyze the characteristics of the acquired respiration signals.

## 3. Detection of Existence of Abnormal Status

**Figure 3:**
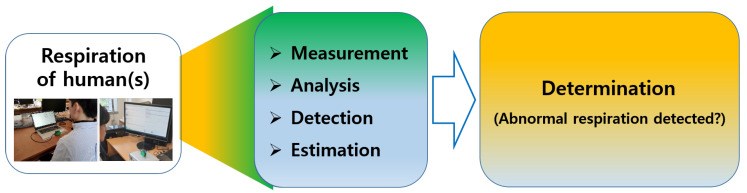
This paper’s goal was to provide a method to detect human respiratory status using a UWB radar sensor.

This section details the proposed approach for detecting and classifying abnormal respiration. The overall flow of detection of abnormal respiration is depicted in Figure 3. Human respiration was measured using a UWB radar, and signal analysis could be performed in diverse domains (in this paper, in the case of a single input, *Z*-domain was used for analysis on the basis of a pole-zero plot. Locations of poles and zeros provided information to analyze and classify characteristics of received data). On the basis of analysis results in various domains, determination of the existence of abnormal respiration was carried out. This is different from other work that chiefly dealt with the accurate measurement of the respiratory rate itself.

In this work, similar to other work dealing with respiration data, the UWB radar received a respiration signal from a human, and the varying distance by inhalation and exhalation visualized the state of respiration. In the case of normal respiration, human breathing generally has a low-frequency component because the movement of the chest surface from respiration is gradually performed. Contrary to normal respiration, abnormal (apnea) may include a high-frequency component due to the rapid movement of the chest surface, i.e., exhalation and inhalation (including no exhalation or inhalation) being carried out over a very short period. The proposed approaches are discussed with two cases, with a single and with multiple respiration signals. The former deals with the classification of respiratory status given one respiration signal. In other words, given a respiration signal, the respiration status of the next input can be determined. Section 3.1 focuses on the determination of respiratory status given a respiration signal, and status estimation is based on *Z*-transform, i.e., positions of zeros and poles. In Section 3.2, we estimate respiration status with multiple-input respiration signals that are simultaneously used as inputs. Among multiple-input signals, we assumed that any input information was not provided, and the existence of abnormal respiration was detected using high-pass filter by varying the cut-off frequency. The high pass filter with cut-off frequency $f_{c}$ is written as

(1) $$H_{hpf}\left(f\right)=1,\;\left(-f_{c}\geq f\geq f_{c}\right),$$

where $H_{hpf}\left(f\right)$ is the Fourier transform of the high-pass filter. In this paper, we used different notations for signals in the cases of a single and of multiple inputs because the proposed approaches for the detection of abnormal respiration are different from each other. In the case of a single input, to clarify input contributions, $A_{j}\left(t\right)$ or $B_{i}\left(t\right)$ was used. In the case of multiple inputs, $R_{i}\left(t\right)$ was used to represent respiration signals (i∈N in both cases). Respiration data were written as

$$\begin{array}{ccc} A_{j}\left(t\right) & = & [A_{j}\left(1\right),A_{j}\left(2\right),\ldots \ldots ,A_{j}\left(N-1\right),A_{j}\left(N\right)],\\ B_{i}\left(t\right) & = & [B_{i}\left(1\right),B_{i}\left(2\right),\ldots \ldots ,B_{i}\left(N-1\right),B_{i}\left(N\right)],\\ R_{i}\left(t\right) & = & [R_{i}\left(1\right),R_{i}\left(2\right),\ldots \ldots ,R_{i}\left(N-1\right),R_{i}\left(N\right)], \end{array}$$

In this paper, input respiration data were analyzed in the *Z* -domain when a single respiration signal was classified. In other cases, multiple-input respiration signals were used as input, and data were analyzed in the time domain by applying a high pass filter with varying cut-off frequency.

### 3.1. Case 1: Single Respiration Signal

**Figure 4:**
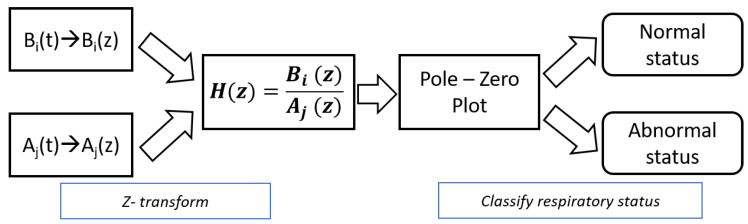
Overall flow of approach to detect respiratory status ($A_{j}\left(z\right)$) given one respiration ($B_{i}\left(z\right)$).

Respiration signals are represented as $A_{i}\left(t\right)$ or $B_{i}\left(t\right)$ in this section (in the time domain), and the *Z* transform of the signals was assumed to exist. *t* is defined as an index of discrete time, and x(t) is defined as a signal in the time domain (*t* is considered discrete time). Then, we can write the equation as follows.

(2) $$\Sigma_{t=-\infty }^{+\infty }\left|x\left(t\right)r^{-t}\right|<\infty ,$$

where *r* represents the region of convergence (ROC) of X(z), which is a *Z* transform of x(t). Then, respiration data $A_{j}\left(t\right)$ and $B_{i}\left(t\right)$, each of which can be analyzed in the *Z* domain, and their *Z* transform is $A_{j}\left(z\right)$ and $B_{i}\left(z\right)$, respectively. In this section, we propose the approach for detecting the existence of apnea given a known respiratory status that is normal or abnormal. In other words, this section deals with the detection of apnea (abnormal respiration) when single-input respiration is used as input. Figure 4 depicts the flow diagram of the proposed approach to classifying respiratory status to detect the existence of abnormal respiration in the *Z* domain. Given respiration $B_{i}\left(t\right)$, the proposed approach determines the status of next-input respiration $A_{j}\left(t\right)$ (either normal or abnormal status).

In particular, the proposed approach provides information about the respiratory status of $A_{i}\left(t\right)$ given $B_{j}\left(t\right)$ by analyzing the signals in *Z* domain. ($A_{i}\left(z\right)$ and $B_{j}\left(z\right)$ are the *Z* transform of $A_{i}\left(t\right)$ given $B_{j}\left(t\right)$, respectively). Classification of respiratory status is performed on the basis of the locations of the poles and zeros of $H_{ij}\left(z\right)$ that depend on whether $A_{i}\left(z\right)$ and $B_{j}\left(z\right)$ are in the same status or not. If respiration is normal, the shape of a signal is similar to a sinusoidal waveform; if not, the shape of a signal has constant (or rapid variation of signal values) values for a few seconds. Since the states of signals directly affect the locations of poles and zeros, we fully exploited the characteristics of $H_{ij}\left(z\right)$.

(3) $$H_{ij}\left(z\right)=\frac{B_{i}\left(z\right)}{A_{j}\left(z\right)},\;i=0,1,\;j=0,1.$$

Given $B_{i}\left(z\right)$, the characteristics of $A_{j}\left(z\right)$ enable us to classify the respiratory status of $A_{j}\left(z\right)$. In practice, in a vehicle, the respiratory status of a driver is given, and the respiration of another person (this person is usually located next to the driver) can be monitored in real time. If both $A_{i}\left(z\right)$ and $B_{j}\left(z\right)$ have the same status, the pole-zero plot of $\frac{A_{i}\left(z\right)}{B_{j}\left(z\right)},\left(i=j\right)$ is significantly different than that of $\frac{A_{i}\left(z\right)}{B_{j}\left(z\right)},\left(i\neq j\right)$. Thus, it is sufficient to classify the respiratory status between two input respiration signals. However, this approach enables us to know discrepancies of respiratory status and whether a new input is abnormal respiratory status based on signal analysis in *Z* domain. This result is, hence, enough to provide information about the existence of abnormal status in a real environment and enables us to prepare or properly manage critical or dangerous conditions a priori. In Equation (3), $A_{0}\left(z\right)$ and $A_{1}\left(z\right)$ were interpreted as normal and abnormal respiration signals, respectively, and $B_{0}\left(z\right)$ and $B_{1}\left(z\right)$ were also interpreted as normal and abnormal, respectively. A set of input signals are any combination of respiratory status, i.e., there are four possible inputs, as shown in Table 1.

The detection of the existence of abnormal respiration is performed by analyzing the pole-zero plot of $\frac{B_{i}\left(z\right)}{A_{j}\left(z\right)}$. Location distributions of poles and zeros vary corresponding to the different conditions of $A_{j}\left(z\right)$ and $B_{i}\left(z\right)$. In the present work, i=j means two inputs are in the same respiratory status, and i≠j means two inputs are in different status. Given a respiratory status, e.g., $B_{i}\left(z\right)$, the status of input respiration $A_{j}\left(z\right)$ generates different pole-zero distribution of $H_{ij}\left(z\right)$, as shown in Section 4. For instance, given normal respiratory status $B_{0}\left(z\right)$, the pole-zero distributions of $\frac{B_{0}\left(z\right)}{A_{0}\left(z\right)}$ and $\frac{B_{0}\left(z\right)}{A_{1}\left(z\right)}$ showed significant differences in pole-zero distribution. Similarly, given $B_{1}\left(z\right)$, $\frac{B_{1}\left(z\right)}{A_{0}\left(z\right)}$ and $\frac{B_{1}\left(z\right)}{A_{1}\left(z\right)}$ showed different distributions of pole-zero plots.

### 3.2. Case 2: Multiple Respiration Signals

**Figure 5:**
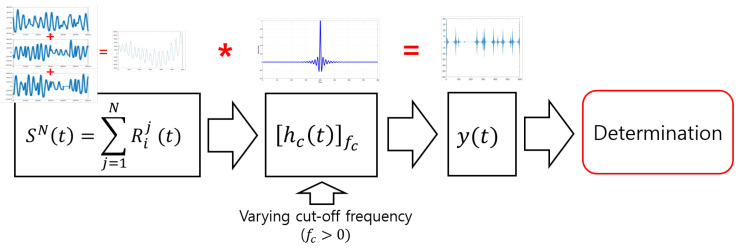
Overall work flow for detecting existence of abnormal respiration with multiple inputs.

This section discusses more complicated and practical aspects in terms of the number of input respiration signals. Multiple respiration signals were used as input, and the existence of an abnormal respiratory state was then detected. This section considers two cases, of two and of three respiration signals as inputs. Figure 5 shows the overall work flow for the determination of respiratory status with multiple inputs.

The *j*th original respiration signal and the summation of all original signals are represented as $R_{i}^{j}\left(t\right)$; (i∈(0,1) represents indices of normal (i=0) or abnormal (i=1) respiration) and $S^{N}\left(t\right)$, respectively. *N* is the number of input respiration signals, each of which is either normal or abnormal. $S^{N}\left(t\right)$ was used as input, and the observed output was y(t), written by

(4) $$y\left(t\right)=S^{N}\left(t\right)★h_{c}{\left(t\right)|}_{f_{c}},$$

where x★y is the convolution of *x* and *y* that can be any type of datum, $h_{c}{\left(t\right)|}_{f_{c}}$ represents a filter with cut-off frequency $f_{c}$ (Hz). In this paper, we employed a high-pass filter, and unit (Hz), which is not important here, was omitted. If analyzed in the frequency domain, Equation (4) can be rewritten as

(5) $$Y\left(f\right)=S^{N}\left(f\right)\times H_{c}{\left(f\right)|}_{f=f_{c}},$$

where Y(f), $S^{N}\left(f\right)$, and $H_{c}{\left(f\right)|}_{f=f_{c}}$ are the Fourier transform of y(t), $S^{N}\left(t\right)$, and $h_{c}{\left(t\right)|}_{f_{c}}$, respectively. $S^{N}\left(t\right)$ contains high-frequency components if at least one instance of apnea (abnormal respiration) is included. As shown in Figure 8, there are parts that have rapid variations of signal amplitude. By varying the cut-off frequency of $h_{c}{\left(t\right)|}_{f_{c}}$, corresponding output y(t) has distinguishable characteristics in time domain. The case of N=2 generated three combination of inputs, i.e., normal- and normal-, normal- and abnormal-, and abnormal- and abnormal-respiration signals. The case of N=3 generated four different combinations, i.e., containing one, two. or three abnormal-respiration signals.

## 4. Simulation Results

This section presents simulation results to substantiate the proposed approach. Simulations were conducted using a UWB radar that was connected to a PC, monitoring and measuring the shape or positional variation of the chest surface owing to respiration (inhalation and exhalation). Respiration signals were acquired by adults (ages from 20 to 40). The radar operated at 8.2 GHz, sampling rate was 10 (per second), and respiration was measured in an iterative manner (30 iterations). Specifications of the UWB radar used for the experiments are described in Table 2.

**Figure 6:**
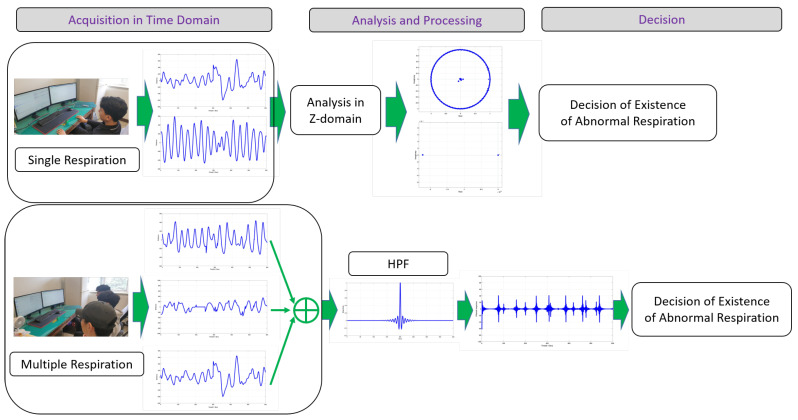
Overall experiment procedures of data acquisition, analysis, processing, and decision for existence of abnormal respiration.

In the experiments, the UWB radar was on a desk, and the tested person, who was breathing normally, was about 30 cm from the radar sensor. In the case of normal and abnormal respiration, a person was breathing sitting on a chair, and the radar was in front of them. This pose was for all cases, stable, speaking, and apnea environments. Particularly in the case of normal respiration while speaking, a person was reading a book, located near the radar, and respiration was acquired by the radar. For the experiments, seven people (male and female) whose ages were in the range from twenty to forty conducted breathing activities. Figure 6 depicts the experiment setup and the scenario of acquisition, analysis, processing, and decision.

**Figure 7:**
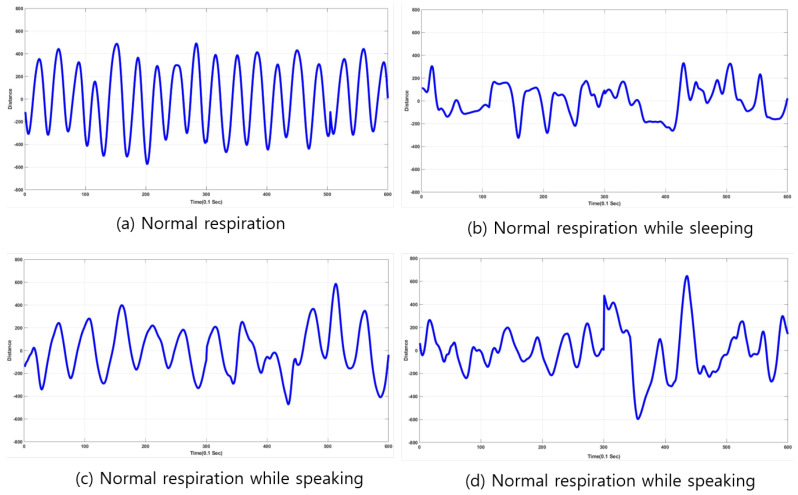
Examples of normal respiration signals. (**a**) Normal respiration in stable environment. (**b**) Normal respiration while sleeping. (**c**,**d**) Normal respiration while speaking.

**Figure 8:**
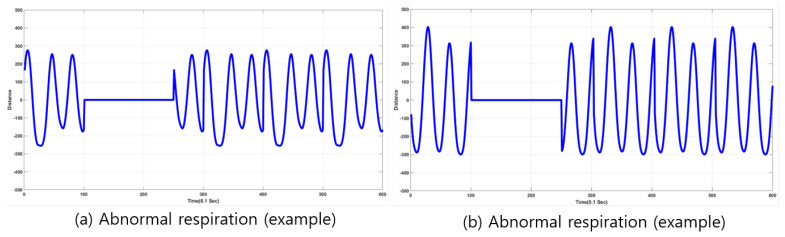
(**a**,**b**) Two examples of abnormal respiration, apnea status.

Examples of respiration signals are depicted in Figure 7 and Figure 8. The examples are composed of normal- and abnormal-respiration signals. An abnormal-respiration signal showed a partial constant of the magnitude representing nonvarying distance between radar and chest surface. Normal respiration included normal respiratory status in a stable environment (no activity and sitting on a chair) while speaking or sleeping.

**Figure 9:**
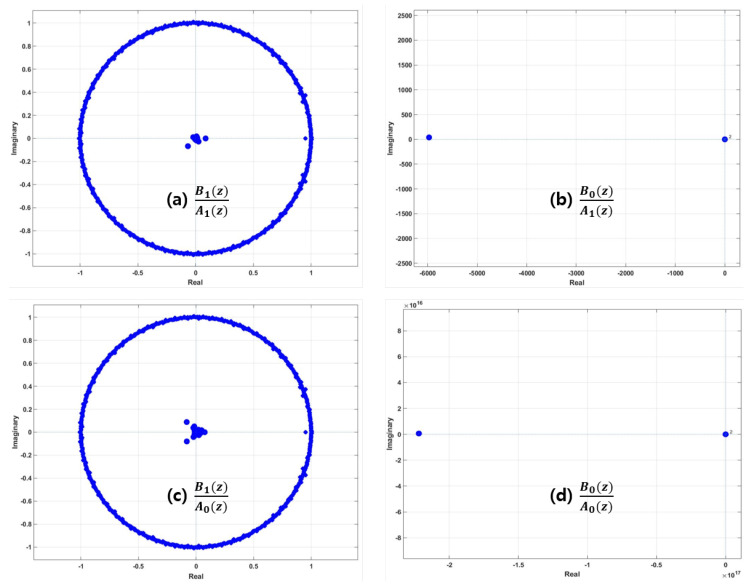
Examples of classification of respiratory status of $A_{j}\left(t\right)$ given $B_{i}\left(t\right)$ using Z-transform. (**a**,**c**) Respiratory status of $A_{j}\left(z\right)$ given abnormal respiration $B_{1}\left(z\right)$. (**b**,**d**) Respiratory status of $A_{j}\left(z\right)$ given normal respiration $B_{0}\left(z\right)$.

In the case of a single input, i.e., input was either a normal- or an abnormal-respiration signal, and each signal was analyzed in *Z*-domain. The positions of zeros and poles provided information to classify respiratory status. This approach is very simple because normal- and abnormal-respiration signals have different positions of zeros and poles leading to clear classification from mathematical and visual perspectives. Results of the Z-transform of $\frac{A_{i}\left(z\right)}{B_{j}\left(z\right)}$ for four cases are shown in Figure 9.

**Figure 10:**
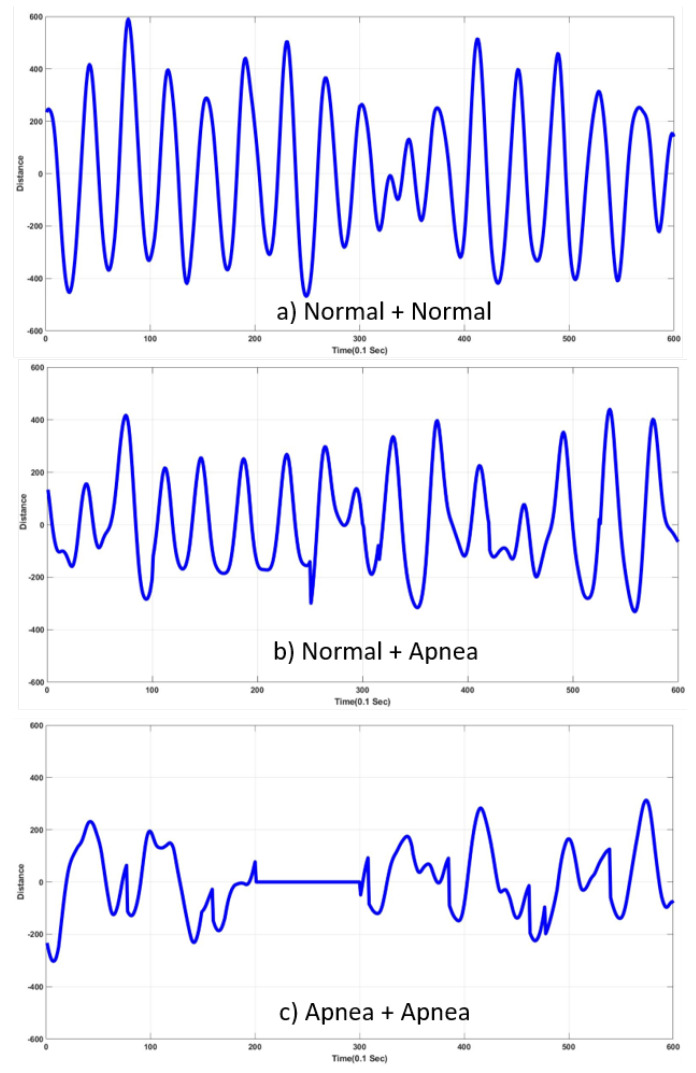
Examples of input signals—addition of two respiration signals. (**a**) $S_{1}^{2}\left(t\right)$; (**b**) $S_{2}^{2}\left(t\right)$; (**c**) $S_{3}^{2}\left(t\right)$.

In the cases of multiple respiration, experiments were conducted using two and three respiration signals, respectively. The former used input signals as a summation of two respiration signals, and the latter used input as the summation of three respiration signals. The addition was not conducted online due to difficulties of the experiment setup and of the simultaneous acquisition of multiple signals. When using two signals as inputs, we had three possible cases of summation:

(6) $$\begin{array}{ccc} S_{1}^{2}\left(t\right) & = & R_{0}^{1}\left(t\right)+R_{0}^{2}\left(t\right), \end{array}$$

(7) $$\begin{array}{ccc} S_{2}^{2}\left(t\right) & = & R_{0}^{1}\left(t\right)+R_{1}^{2}\left(t\right), \end{array}$$

(8) $$\begin{array}{ccc} S_{3}^{2}\left(t\right) & = & R_{1}^{1}\left(t\right)+R_{1}^{2}\left(t\right), \end{array}$$

where $S_{j}^{2}\left(t\right)$, (i=1,2,3), $R_{0}^{1}\left(t\right)$, and $R_{1}^{2}\left(t\right)$ represent the summation of respiration signals, and normal- and abnormal-respiration signals, respectively (to avoid confusion with the case of single respiration, we used $R_{j}^{i}\left(t\right)$ to represent a signal here, and $R_{0}^{1}\left(t\right)$ and $R_{1}^{2}\left(t\right)$ to represent normal and abnormal signals. Equations (9)–(12) could be similarly interpreted as Equation (6) is. Inputs were either normal or abnormal respiration as shown in Figure 7 and Figure 8. Any two respiration signals were used as input, and $R_{i}^{1}\left(t\right)$ and $R_{i}^{2}\left(t\right)$ (i∈[0,1]) were added. Examples of $S_{j}^{2}\left(t\right)$ (j∈[1,2,3]) are shown in Figure 10.

**Figure 11:**
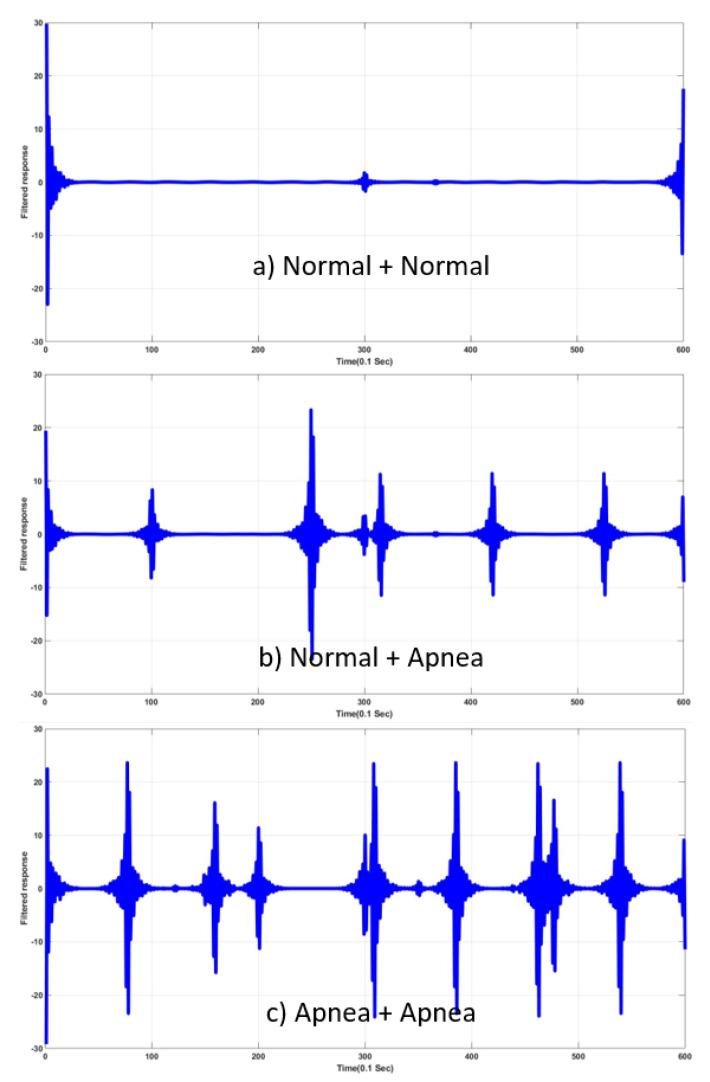
Responses to filter with varying cut-off frequency providing information about existence of abnormal respiration. (**a**) $S_{1}\left(t\right)*h_{c}\left(t\right)$. (**b**) $S_{2}\left(t\right)*h_{c}\left(t\right)$. (**c**) $S_{3}\left(t\right)*h_{c}\left(t\right)$.

Once input $S^{N}\left(t\right)$ was acquired, high-pass filter (HPF) with varying cut-off frequency applied to the input, and responses to the filter, $S^{N}\left(t\right)★h_{c}{\left(t\right)|}_{f_{c}}$ provided information to determine the existence of an abnormal-respiration signal between the two signals. Plots of $S^{N}\left(t\right)★h_{c}{\left(t\right)|}_{f_{c}}$ are shown in Figure 11.

**Figure 12:**
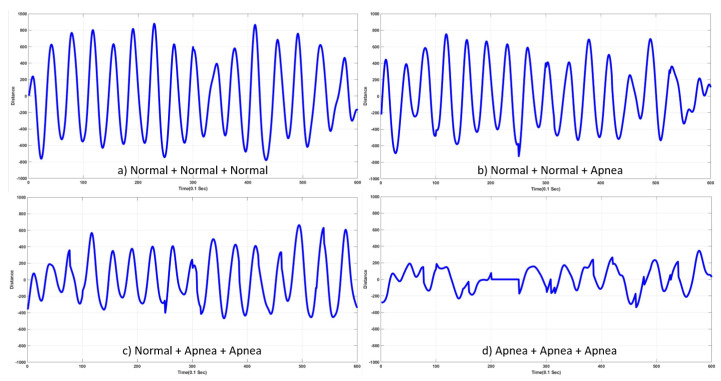
Examples of input signals—addition of three input respiration signals. (**a**) $S_{1}^{3}\left(t\right)$; (**b**) $S_{2}^{3}\left(t\right)$; (**c**) $S_{3}^{3}\left(t\right)$; (**d**) $S_{4}^{3}\left(t\right)$.

Abnormal respiration could be detected when a combination of three respiration signals were used:

(9) $$\begin{array}{ccc} S_{1}^{3}\left(t\right) & = & R_{0}^{1}\left(t\right)+R_{0}^{2}\left(t\right)+R_{0}^{3}\left(t\right), \end{array}$$

(10) $$\begin{array}{ccc} S_{2}^{3}\left(t\right) & = & R_{0}^{1}\left(t\right)+R_{0}^{2}\left(t\right)+R_{1}^{3}\left(t\right), \end{array}$$

(11) $$\begin{array}{ccc} S_{3}^{3}\left(t\right) & = & R_{0}^{1}\left(t\right)+R_{1}^{2}\left(t\right)+R_{1}^{3}\left(t\right), \end{array}$$

(12) $$\begin{array}{ccc} S_{4}^{3}\left(t\right) & = & R_{1}^{1}\left(t\right)+R_{1}^{2}\left(t\right)+R_{1}^{3}\left(t\right), \end{array}$$

where $R_{0}^{j}\left(t\right)$ and $R_{1}^{j}\left(t\right)$ (j∈(1,2,3))represent normal and abnormal respiration, respectively, and $S_{1}^{3}\left(t\right)$ and $S_{4}^{3}\left(t\right)$ include all normal- and abnormal-respiration signals, respectively. $S_{2}^{3}\left(t\right)$ and $S_{3}^{3}\left(t\right)$ include one and two abnormal respiration signal(s), respectively. Detection of abnormal signals was desired in $S_{2}^{3}\left(t\right)$, $S_{3}^{3}\left(t\right)$, and $S_{4}^{3}\left(t\right)$. Figure 12 shows input signals as described in Equations (9)–(12).

**Figure 13:**
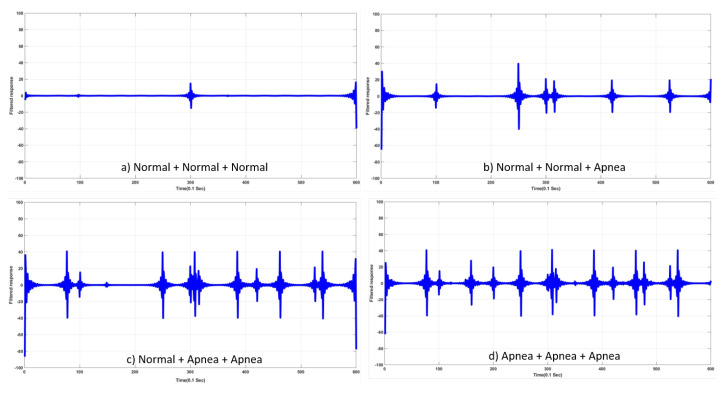
Examples of outputs that were filtered responses by varying cut-off frequency of high-pass filter. (**a**) $S_{1}^{3}*h_{c}\left(t\right)$. (**b**) $S_{2}^{3}*h_{c}\left(t\right)$. (**c**) $S_{3}^{3}*h_{c}\left(t\right)$. (**d**) $S_{4}^{3}*h_{c}\left(t\right)$

Similar to the case of two inputs, once input $S_{i}^{j}\left(t\right)$ was acquired, a high-pass filter (HPF) with varying cut-off frequency was applied to the input, and responses to the filter, $S_{i}^{j}\left(t\right)*h_{c}\left(t\right)$ provided information to determine the existence of an abnormal-respiration signal between the two signals. Plots of $S_{i}^{j}\left(t\right)*h_{c}\left(t\right)$ are shown in Figure 13.

Accuracy of classification was calculated as classification-success rate as follows.

(13) $$\begin{array}{c} R_{A}=\frac{N_{RA}}{N_{A}}, \end{array}$$

(14) $$\begin{array}{c} R_{N}=\frac{N_{RN}}{N_{N}}, \end{array}$$

where $R_{A}$ and $R_{N}$ represent the accurate detection of existence of abnormal and normal signals (the case of Equations (8) and (12), respectively). $N_{A}$ is the total number of input signals ($S_{i}^{j}\left(t\right)$) that included at least one instance of abnormal respiration, and $N_{RA}$ is the number of results that accurately detected the existence of abnormal-respiration signals (not the number of abnormal signals). Similarly, $N_{N}$ and $N_{RN}$ are the total number of input signals that included only normal respiration, and the number of accurate determinations of the existence of normal-respiration signals (not the number of normal-respiration signals), respectively. For example, in the case of Equation (13), let us consider three inputs, one abnormal- and two normal-respiration signals. If the result of the algorithm says "abnormal respiration exists in the input", the algorithm was considered to be correct, i.e., $N_{RA}=N_{RA}+1$. Accuracy is shown in Table 3. Previous work on investigating respiratory status usually focused on the accurate measurement of respiratory rate, and the detection of respiratory patterns usually deals with a single respiration signal. Although it is difficult to directly compare the proposed work to existing work, we compared the detection success rate of abnormal respiration signals in the case of a single input.

## 5. Conclusions

This paper presented approaches for the detection of abnormal respiration. Human respiration states are a good predictor and indicator to assess physical and physiological status. In particular, this paper dealt with cases of single- and multiple-input respiration signals on the basis of noncontact-based measurements. In the case of single-input respiration, signals were analyzed in the Z domain, and the locations of poles and zeros provided information about respiration status, enabling us to classify respiration status. Given one respiration signal that was already acquired by UWB radar, the status of the next input respiration could be determined by analyzing the locations of poles and zeros. Multiple inputs were used, namely, two and three respiration signals, and the summation of all of the inputs was used as input to our system. A high-pass filter was applied to the input, varying cut-off frequency, and output was a response to the filter to provide information about the existence of an abnormal-respiration signal. The proposed approach showed promising results for the determination of respiratory status in multiple-input signals. The present work can be applied to vehicular environments or other ones that require real-time and long-term continuous monitoring and estimation of human respiratory status. In the future, the number of abnormal signals included in the multiple inputs will be estimated on the basis of signal-processing techniques with machine-learning (including neural-network) algorithms.

## Figures and Tables

**Table 1:** Notations used to categorize respiratory status.

$A_{j}\left(z\right)$	$B_{i}\left(z\right)$
Normal ($A_{0}\left(z\right)$)	Normal ($B_{0}\left(z\right)$)
Normal ($A_{0}\left(z\right)$)	Abnormal ($B_{1}\left(z\right)$)
Abnormal ($A_{1}\left(z\right)$)	Normal ($B_{0}\left(z\right)$)
Abnormal ($A_{1}\left(z\right)$)	Abnormal ($B_{1}\left(z\right)$)

**Table 2:** Specification of UWB radar [27].

Components	Specification
Frequency	4.1–10.3 GHz
Bandwidth	1.7–3.1 GHz
TX peak power	−40 dBm/50 MHz
TX min power	−60 dBm/MHz
Power consumption	180 mA
Package	40×45 mm
Motion range	10 m
Respiration range	5 m

**Table 3:** Accuracy of detection and classification.

Category	Accuracy (Proposed) (%)	Kim et al. [28]	Miao et al. [29]
Single input	67%	93%	85.8%
Two inputs	87%	93%	85.8%
Three inputs	93%	93%	85.8%
